# Evaluating the Potential of Using *Spodoptera litura* Eggs for Mass-Rearing *Telenomus remus*, a Promising Egg Parasitoid of *Spodoptera frugiperda*

**DOI:** 10.3390/insects12050384

**Published:** 2021-04-26

**Authors:** Wanbin Chen, Yuyan Li, Mengqing Wang, Jianjun Mao, Lisheng Zhang

**Affiliations:** State Key Laboratory for Biology of Plant Diseases and Insect Pests, Institute of Plant Protection, Chinese Academy of Agricultural Sciences, Beijing 100193, China; chenwb24@126.com (W.C.); lyy129@126.com (Y.L.); mengqingsw@163.com (M.W.); Maojianjun0615@126.com (J.M.)

**Keywords:** biological control, development, alternative host, temperature, thermal requirements, mass-rearing, *Telenomus remus*, *Spodoptera litura*, *Spodoptera frugiperda*

## Abstract

**Simple Summary:**

*Telenomus remus* (Nixon) is an effective egg parasitoid for controlling *Spodoptera frugiperda* (J. E. Smith)*,* which is a major destructive agricultural pest. Currently, this parasitoid is reared on *Corcyra cephalonica* (Stainton) eggs in several countries. However, previous studies carried out in China have reported that it cannot parasitize in *C. cephalonica* eggs. Meanwhile, those works have indicated that *Spodoptera litura* (Fabricius) can potentially be used as an alternative host. In order to evaluate this potential, our study compared the development and parasitism ability of *T. remus* on the eggs of *S. frugiperda* and *S. litura* at different temperatures in a laboratory. We found that *S. litura* eggs are more advantageous as an alternative host for the mass-rearing of parasitoid when compared with *S. frugiperda* eggs. Our results provide a more specific basis and reference for the large-scale production and low temperature storage of *T. remus*.

**Abstract:**

Although *Telenomus remus*, a promising parasitoid of *Spodoptera frugiperda*, had been successfully reared on the eggs of *Corcyra cephalonica* in some countries, reports from China have argued that it is infeasible. Notably, studies from China have indicated that *Spodoptera litura* eggs could be a candidate host. Therefore, to further evaluate the potential of using *S. litura* eggs as hosts, we compared the development and parasitism of *T. remus* on the eggs of *S. frugiperda* and *S. litura* at temperatures between 20–32 °C. Our results showed that *T. remus* developed successfully on both host eggs at all of the tested temperatures, and the developmental duration and thermal requirements at each stage were similar between the two host species. The number of parasitized eggs was greater for *S. litura* than for *S*. *frugiperda*. Meanwhile, the emergence rate exceeded 86.6%, and it was significantly higher for *S. litura* than that for *S*. *frugiperda,* except at 29 °C. This study is the first time estimating the thermal requirements of *T. remus* at each stage. Moreover, we also recorded the morphological characteristics of *T. remus* at each stage. Our results demonstrate that *S. litura* eggs are more suitable than *S. frugiperda* eggs as an alternative host for the mass-rearing of *T. remus* in China. Understanding the thermal requirements and biological parameters contributes greatly to predicting the generation time and providing a reference for the mass-rearing and storage of the parasitoid.

## 1. Introduction

The fall armyworm *Spodoptera frugiperda* (J. E. Smith; Lepidoptera: Noctuidae) is a major destructive agricultural pest worldwide [1]. *S. frugiperda* is a polyphagous pest that can feed on 353 different plant species, including maize, rice, wheat, and soybean, and it especially prefers maize [2]. *S. frugiperda* poses a substantial threat to agricultural production and food security [1]. For example, if no control methods are taken, the annual maize yield losses caused by *S. frugiperda* are expected to reach 8.3 million to 20.6 million tonnes within the 12 main maize-producing countries of Africa [3].

As of late 2019, *S. frugiperda* was established in Southern China, and now persists all year round [4]. Owing to China’s diverse geography, complex crop planting structure, and abundant plant resources, controlling this pest constitutes a formidable, long-term challenge [4]. The application of insecticides is the most effective strategy for controlling *S. frugiperda*; however, excessive and/or inappropriate application has led to serious issues, such as evolution of resistance, elimination of beneficial insect, and environmental pollution [3,5]. To meet the need for the sustained development of agriculture in China, one ecologically friendly strategy for controlling *S. frugiperda* is via biological control using natural enemy insects and entomopathogens [6,7].

More than 290 natural enemy resources of *S. frugiperda* have been reported [8,9]. Among these agents, the egg parasitoid *Telenomus remus* (Nixon; Hymenoptera: Platygastridae) is one of the most commonly used effective species for controlling *S. frugiperda* [10,11,12]. The body length of the parasitoid adult is only 0.5–0.6 mm and usually presents as shiny black in color. *T. remus* lay their eggs inside the developing host, and the larva consume the nutrition of the host to satisfy its development [12]. When emerging, the parasitoid adult will chew a small hole in the host eggs and crawl out. The successful application of *T. remus* in biological control programs depends primarily on its high parasitism and capacity to parasitize the inner layer of insect egg masses [12]. In Brazil, for example, the inhibition of *S. frugiperda* egg masses by *T. remus* reached 54–99% in maize, cotton, and soybean fields, indicating the highly efficient control of this insect [13].

Although *T. remus* displays considerable potential for controlling *S. frugiperda*, it has proven to be challenging to find a suitable host for the mass-rearing of *T. remus* at an affordable cost in China [14]. Research has shown that *T. remus* can be reared successfully on the eggs of *Corcyra cephalonica* (Stainton) [15,16]. However, our preliminary experiments and other studies [14] conducted in China have indicated that *T. remus* cannot parasitize and develop in *C. cephalonica* eggs. Since the invasion of *S. frugiperda* in China in early 2019, researchers have reared *T. remus* using its natural host *S. frugiperda* eggs [14]. However, *S. frugiperda* is highly cannibalistic, making it difficult to sustain an affordable, large-scale rearing system in terms of both time and resources. Therefore, it is urgent to identify a suitable alternative host egg in China. Studies have shown that *Spodoptera litura* (Fabricius) eggs could potentially serve as an alternative host for the mass-rearing of *T. remus* [12,17]. Furthermore, *S. litura* larvae exhibit little or no cannibalism [18].

Understanding the relationship between development and temperature enables the prediction of the number of insect generations annually in a certain area, which is important to determine the release time of natural enemies [19]. Several reports have been published pertaining to the temperature requirements of some insects [19,20]. However, few reports have studied the temperature requirements of *T. remus*, from which the thermal constant (*K*) for complete development is 154.12 degree-days (DD) for males and 158.88 DD for females [21]. Importantly, the thermal requirements for eggs, larvae, prepupae, and pupae remain unclear for *T. remus*.

It is clear that an evaluation of the development and parasitism capacity of *T. remus* on the eggs of *S. frugiperda* or *S. litura* at different temperatures would be conducive to providing sufficient evidence to confirm the potential of *S. litura* eggs as a suitable alternative host for the mass-rearing of *T. remus*. The data obtained would also provide fundamental knowledge for biological control programs using *T. remus* for controlling *S. frugiperda* in China. Therefore, we compared the effect of host species, namely *S. frugiperda* and *S. litura*, on the development, survival, and parasitism of *T. remus* at different temperatures, and determined the developmental threshold temperature (*T*_0_), thermal constant, and correlative biological parameters of *T. remus*.

## 2. Materials and Methods

### 2.1. Insect Culture

*T. remus* was obtained from colonies kept at the maize pest laboratory at the Institute of Plant Protection, Chinese Academy of Agricultural Sciences. Parasitized egg masses of *S. frugiperda* were originally collected from maize fields in Jinhua, Zhejiang Province, China, in 2019. The colony of *T. remus* was reared on those egg masses under a climatic incubator (26 ± 1 °C, 70 ± 5% of relative humidity (RH), 14 h:10 h light (L)/dark (D)).

The *S. frugiperda* larvae were collected from maize fields in Kunming, Yun’nan Province, China, in 2019. The population was maintained for over 10 generations in a climatic incubator at 28 ± 1 °C, 60 ± 5% RH, 16 h:8 h L/D. The first to third instar larvae were reared together in plastic cages (34 cm × 22 cm × 4 cm) containing fresh maize leaves. To avoid cannibalism, the fourth to sixth instar larvae were separately fed an artificial diet [22] in cylindrical plastic containers (3 cm in height × 5 cm in diameter). For the purpose of mating and oviposition, the adults were reared in cylindrical wire-mesh cages (28 cm in height × 24 cm in diameter), the inner surface and the upper opening of which were covered with wax paper and wet gauze to provide an oviposition substrate and retain moisture, respectively. Adults were fed a 20% honey solution, which was replaced daily. The wax paper and gauze with egg masses were collected every morning and were used for the following experiments.

The *S. litura* was obtained from the Institute of Plant Protection, Jilin Academy of Agricultural Sciences, Jilin Province, China. The same aforementioned conditions and methods were used to rear this species, except that the larvae were reared in groups instead of individually (as with *S. frugiperda*), using a specific artificial diet [23].

### 2.2. Effects of Temperature and Host Species on T. remus Development

All of the trials were conducted in a climatic incubator (RXZ-500, Ningbo Jiangnan Instrument Factory, Zhejiang Province, China). A 14L:10D photoperiod was used in all of the incubators and the humidity varied between 65 and 75% RH.

Previous studies have shown that the mortality of *T. remus* was about 87% when reared on *S. frugiperda* eggs at 15 °C, and even reached 100% at 35 °C [21]. Therefore, five constant temperatures (20, 23, 26, 29, and 32 ± 1 °C) were selected to estimate the effects of temperature on the development and parasitism of *T. remus* reared on the eggs of *S. frugiperda* or *S. litura* in present study. Fresh egg masses of *S. frugiperda* or *S. litura* (<24 h old) were exposed to newly emerged (<12 h old) and mated *T. remus* adults for 1 h, after which parasitized eggs were divided equally into five groups that were kept at different temperatures for development. The egg, larvae, prepupae, and pupae stages of *T. remus* were observed via dissection at 4 h intervals under a stereomicroscope, and photos of *T. remus* at each stage were taken (SZX10, Olympus Corporation, Japan). Because the newly deposited eggs were difficult to observe, the egg and the first instar larvae were considered as a single stage, namely “egg-first instar larvae”. The parasitized eggs were checked periodically until emergence, and the duration of each stage (egg-first instar larvae, second instar larvae, prepupae, and pupae) was recorded. There were 50 replicates for each treatment.

### 2.3. Effects of Temperature and Host Species on the Fecundity and Lifespan of T. remus

The inner-surface wax paper for the cages, with approximately 200 fresh eggs of *S. frugiperda* or *S. litura* (<24 h old), was placed inside a transparent plastic tube (10 cm in height × 2.5 cm in diameter) containing a single newly emerged and mated female adult of *T. remus* (<24 h old), with subsequent incubation at various temperatures. The inner surface of the tube contained a drop of 20% honey. The egg-containing wax paper was replaced daily until the death of the female parasitoid, and then the parasitized eggs were moved into a new tube kept under the specific conditions for development until the offspring emerged. During the period in which the *T. remus* larvae developed, the *Spodoptera* caterpillars that hatched from non-parasitized eggs were removed to prevent them feeding on the parasitized eggs. Each experimental trial had at least 20 replicates. The number of parasitized eggs, emergence rate, percentage of female, and lifespan were recorded.

### 2.4. Statistical Analysis

The relationship between the temperature and developmental rate were estimated by linear regression using Equation (1) [24],

Y = a + b × X(1)

where Y is the developmental rate, X is the temperature, and a and b are the estimated parameters of the regression. The thermal constant and developmental threshold temperature were calculated using the parameters: *K* = 1/b and *T*_0_ = −a/b.

The effects of the temperature, host egg species, and the interactions among these factors on developmental duration, number of parasitized eggs, emergence rate, percentage of females, and lifespan were analyzed by two-way ANOVA. The differences among the five different temperatures (20, 23, 26, 29, and 32 °C) were compared using Tukey’s honest significant difference (HSD) tests at a 0.05 level. The differences between the two host egg species (*S. frugiperda* and *S. litura*) were compared using an independent-samples *t*-test. The percentage data were arcsine square-root-transformed to homogenize the variances prior to analysis, whereas the data for female lifespan was log10-transformed to fit a normal distribution. All of the statistical analyses were performed using SPSS version 19.0 (IBM Crop., Chicago, IL, USA) for Windows. The figures were created using GraphPad Prism for Windows (version 8.0) (Graphpad Software, Inc., San Diego, CA, USA).

## 3. Results

### 3.1. Development of T. remus Reared on Eggs of S. frugiperda or S. litura

**Figure 1:**
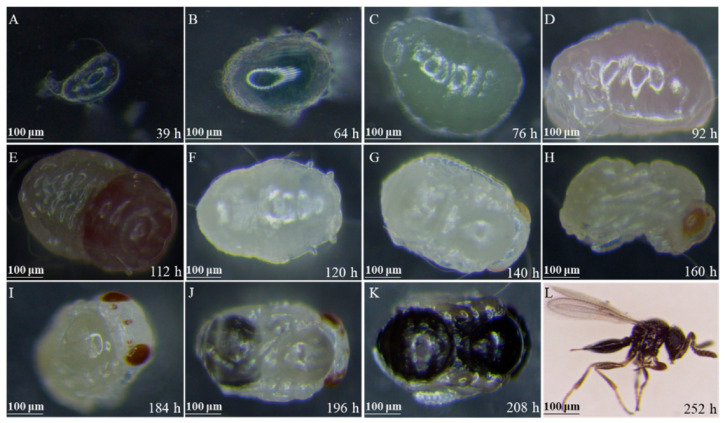
Morphological characters of *T. remus* at the first instar larvae (**A**,**B**), second instar larvae (**C**), prepupae (**D**,**E**), pupae (**F**–**K**), and adult (**L**).

The typical morphological characters regarding the development of *T. remus* reared on *S. frugiperda* eggs at 26 °C are shown in Figure 1, and the duration from egg to adulthood of *T. remus* was ~10 days.

The results showed that *T. remus* was able to develop successfully on the eggs of *S. frugiperda* and *S. litura* at all of the temperatures we tested. However, the developmental duration of *T. remus* at each stage lengthened with the decrease in temperature. The temperature and host species have a significant effect on the duration of the egg-first instar larvae. However, the interaction between the temperature and host species had no effect on the duration (Table 1). At 32 °C, the duration of the egg-first instar larvae was significantly longer for the *S. frugiperda* eggs than the *S. litura* eggs (t = 3.301; df = 98; *p* = 0.001) (Table 2). The duration of both the second instar larvae and prepupae were influenced by the temperature, but not by the host species or their interactions (Table 1). There was no obvious difference in the prepupae duration between 29 °C and 32 °C (Table 2). The temperature, host species, and the interaction between these two factors significantly influenced the developmental duration of both the pupae and generation (Table 1). At 20 °C (pupae: t = −5.589; df = 98; *p* < 0.0001; generation: t = −3.526; df = 98; *p* = 0.0001), 26 °C (pupae: t = 5.300; df = 98; *p* < 0.0001; generation: t = 5.534; df = 98; *p* = 0.0001), 29 °C (pupae: t = −16.081; df = 98; *p* < 0.0001; generation: t = −14.973; df = 98; *p* < 0.0001), and 32 °C (pupae: t = 5.411; df = 98; *p* < 0.0001; generation: t = 9.276; df = 98; *p* < 0.0001), the duration of both the pupae and generation obviously differed between the host species; however, at 23 °C, there was no difference between the host species (Table 2). For each host species, there was an approximately three-fold difference in generation time between 20 °C and 32 °C (Table 2).

### 3.2. Thermal Requirements of T. remus Reared on the Eggs of S. frugiperda or S. litura

**Figure 2:**
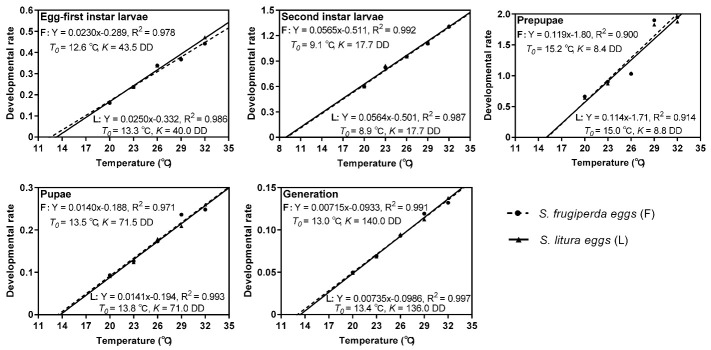
Developmental rate of each stage of *T. remus* reared on eggs of *S. frugiperda* or *S. litura* at different temperatures.

The values *K* and *T*_0_, as well as the regression equations of developmental rate for each developmental stage are shown in Figure 2. The *T*_0_ and *K* of each stage were not significantly different between the host species. The second instar larvae and prepupae presented the lowest *T*_0_ and *K*, respectively. The *K* of the parasitoids was similar when reared on the eggs of *S. frugiperda* or *S. litura*, with 140.0 DD above a threshold of 13.0 °C and 136.0 DD above a threshold of 13.4 °C, respectively. All of the regression equations showed a coefficient of correlation (R^2^) higher than 0.900, demonstrating that the model provided a satisfactory fit for the relationship between the developmental rate and temperature (Figure 2).

### 3.3. Biological Characteristics of T. remus Reared on Eggs of S. frugiperda or S. litura

**Figure 3:**
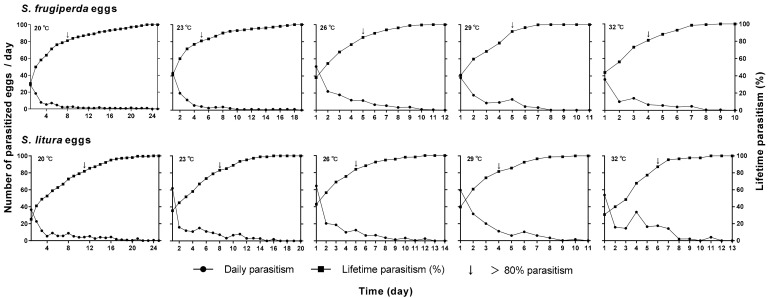
Cumulative and daily parasitism of *T. remus* reared on the eggs of *S. frugiperda* and *S. litura* at different temperatures.

The oviposition rhythm of *T. remus* varied with the temperature and host species, and daily parasitism decreased gradually over the female lifespan. The parasitism capacity was maximal on the first day at all of the tested temperatures (Figure 3). For *S. frugiperda*, the number of parasitized eggs on the first day was 29.3, 43.5, 51, 37.9, and 36.8 at 20, 23, 26, 29, and 32 °C, respectively, and the corresponding values for the *S. litura* eggs were 36.5, 61.9, 64.5, 59.6, and 53.9, respectively, each of which was greater than that for *S. frugiperda*. For the *S. frugiperda* eggs, the time to reach 80% parasitism was 8, 5, 5, 5, and 4 days at the five temperatures, and the corresponding times were 11, 8, 5, 4, and 6 days, respectively, for the *S. litura* eggs (Figure 3).

**Figure 4:**
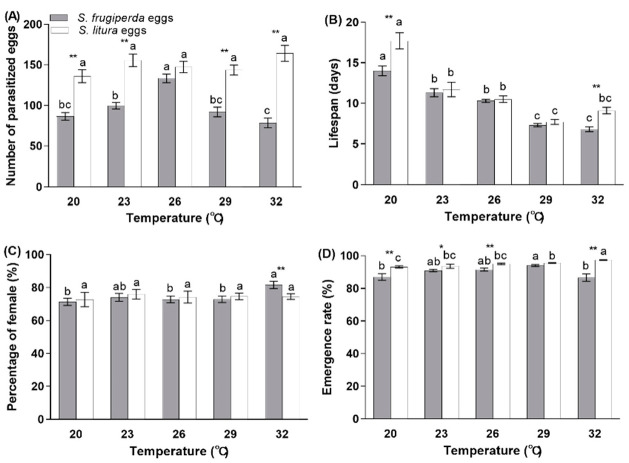
The number of parasitized eggs (**A**), female lifespan (**B**), percentage of females (**C**), and emergence rate (**D**) for *T. remus* reared on eggs of *S. frugiperda* or *S. litura* at different temperatures. Different lowercase letters indicate a statistically significant difference among different temperatures (Tukey’s test, *p* < 0.05). Asterisks indicate a significant difference between two host species (*t*-test, * *p* < 0.05; ** *p*< 0.01).

There was an effect from the temperature and host species, as well as the interaction between these two factors, on the number of parasitized eggs (Table 1). For *S. frugiperda*, the total number of parasitized eggs differed significantly among the various tested temperatures (F = 16.118; df = 4, 151; *p* < 0.0001), with the highest parasitism ability occurring at 26 °C (133.4 eggs); however, for *S. litura*, the parasitism was similar among the various temperatures (from 136.1 eggs at 20 °C to 164.2 eggs at 32 °C). The capacity of *T. remus* to parasitize the eggs of *S. litura* was significantly greater than for the *S. frugiperda* eggs at 20 °C (t = −5.664; df = 52; *p* < 0.0001), 23 °C (t = −7.115; df = 50; *p* < 0.0001), 29 °C (t = −6.015; df = 62; *p* < 0.0001), and 32 °C (t = −7.486; df = 50.973; *p* < 0.0001; Figure 4A).

Both the temperature and host species significantly impacted the lifespan of the parental females, as well as the interaction between the temperature and host species (Table 1). The lifespan of the female adult differed between host species only at 20 °C (t = −2.690; df = 52; *p* = 0.01) and 32 °C (t = −3.203; df = 60; *p* = 0.002). The females that developed on *S. litura* eggs lived longer than those on *S. frugiperda* eggs at 20 and 32 °C, but the lifespans were similar between the two host species within the range of 23–29 °C. The female lifespan decreased continuously from 20 °C to 32 °C, with a range from 14 to 6.8 days (F = 50.893; df = 4, 151; *p* < 0.0001) for *S. frugiperda* eggs and from 17.7 to 7.7 days (F = 25.679; df = 4, 123; *p* < 0.0001) for *S. litura* eggs, respectively (Figure 4B).

There was no effect from the temperature and host species, as well as the temperature and host species interaction, on the number of female offspring (Table 1). The percentage of female progeny only differed between the host species at 32 °C (t = 2.816; df = 60; *p* = 0.007), with a higher offspring sex ratio for *S. frugiperda* eggs. For *S. frugiperda* eggs (F = 3.970; df = 4, 151; *p* = 0.004), the temperature had a significant effect, with fewer female offspring at 20, 26, and 29 °C compared with 32 °C. For the *S. litura* eggs, the percentage of females was similar at different temperatures (Figure 4C).

The temperature and host species, as well as the temperature and host species interaction, had a significant effect on the adult emergence (Table 1). The emergence rate of *T. remus* was higher for *S. litura* eggs than for *S. frugiperda* eggs at 20 °C (t = −2.838; df = 45.573; *p* = 0.007), 23 °C (t = −2.526; df = 50; *p* = 0.015), 26 °C (t = −3.700; df = 47.277; *p* = 0.001), and 32 °C (t = −6.211; df = 35.188; *p* < 0.0001), but not at 29 °C. For the *S. frugiperda* eggs (F = 5.197; df = 4, 151; *p* = 0.001), the emergence rate was the highest at 29 °C (94%); this value was less at 20 °C (87%) and 32 °C (86.6%). For the *S. litura* eggs (F = 13.099; df = 4, 123; *p* < 0.0001), the emergence rate was higher than 93.1% for all of the tested temperatures, with the highest rate at 32 °C (Figure 4D).

## 4. Discussion

Regarding parasitoid developmental duration of each stage, the results obtained in this study indicated that the developmental duration of *T. remus* on the eggs of *S. frugiperda* and *S. litura* is similar at any given temperature, which is similar to the results found by Pomari et al. [25], who studied the egg-to-adult period of *T. remus* reared on the eggs of *S. frugiperda*, *S. albula* (Walker), *S. cosmioides* (Walker), and *S. eridania* (Cramer) over a temperature range of 19–34 °C, who reported a similar developmental time for *T. remus* among the four host eggs. Generally, the parasitoid biological parameters differ depending on the host species [20]. These different traits of host egg species, including the size, surface, and chemical cues of the host egg chorion structure, affect the host suitability for the parasitoid [26,27]. The adaptability of the parasitoid to different host species may be affected by differences in their coevolutionary relationships with each species [28]. The eggs of *S. frugiperda* are 0.4 mm in diameter and 0.42 mm in length [26], whereas *S. litura* eggs are 0.6 mm in diameter [29]. The parasitoid usually showed better development and adaptation on the more suitable host eggs. Consistent with this, the parasitism capacity of *T. remus* was greater for the *S. litura* eggs because of their bigger egg size, which may provide more nutrients and thus affect the parental parasitism and progeny fitness of the parasitoid [30]. Theoretically, an abundant availability of nutrients might permit more than one parasitoid larva to complete development and emergence. However, the phenomenon of a single progeny parasitoid per host eggs, observed in this and other studies [12], might be due to the consequence of parasitoid larva competition for nutrients when two or more parasitoid eggs are laid inside one host egg [12]. Furthermore, the host egg quality has previously been pointed out as a major factor affecting the sex ratio of the parasitoid, particularly for Trichogrammatidae [28]. This might be due to the ability of the parental parasitoid to distinguish the quality of host egg before laying male or female eggs [28]. The offspring represented a similar percentage of females between the eggs of *S. frugiperda* and *S. litura*, indicating a similar quality for the two host species.

Consistent with previous studies [25,31], the development and biological characteristics of *T. remus* were highly affected by temperature. Therefore, exploring the relationship between temperature and biological performance is of great significance for a more comprehensive understanding of the ecology and management of insects [32]. The results from the present study demonstrate that when lengthening the developmental duration, egg-first instar larvae, second instar larvae, prepupae, pupae, and generation, inversely follow a decrease in temperature. Behavioral plasticity may explain the effects of temperature on insect development [33]. Specifically, a high temperature increases the metabolic rate, resulting in a shortened developmental time, which is prolonged at low temperatures [34]. *T. remus* could survive and complete development in the temperature range of 20–30 °C. However, a high mortality was apparent at 34 °C, and at 35 °C, no *T. remus* adult emergence occurred, which showed that the upper limit of temperature for this species may be between 34 °C and 35 °C [21,25].

Temperature is a key factor governing both the metabolic rate and lipid consumption of parasitoids. There is generally an inverse relationship for both factors [35]. Adult fecundity and lifespan are largely dependent on the amount of body lipids that have accumulated during the larval stage, and adult feeding on honey or nectar does not increase the lipid reserves [36]. Thus, temperature may impact the distribution of lipids, which may further affect the balance between lifespan and fecundity. Generally, the oviposition peak of *T. remus* occurs in the first 24 h, regardless of environmental conditions, parasitoid population, or host species, and parasitism gradually decreases thereafter [35,37]. The egg parasitoids of the genus *Trichogramma* display the same characteristics, which might imply that the females need to search an appropriate host promptly on which to lay their eggs after emergence [30,38]. Overall, it is must be considered whether the parasitism activity is concentrated during a certain timeframe, namely, soon after emergence or continuously throughout adulthood, which may affect the performance of parasitoid in the field [31,35].

It was reported previously that *T. remus* presents poor parasitism at extreme temperatures [31]. The decreased fecundity of female parasitoids at high temperatures is a consequence of a reduced lifespan [39], which implies that temperature optimization is vital for the build-up of a large-scale population. In addition, eggs lose water faster at a high temperature, which may explain the observed decrease in the parasitism capacity [31]. The influence of temperature on the lifespan of female parasitoids was inversely related, as reported by Bueno et al. and Pomari et al. [31,35], probably because both the metabolic activity and energy expenditure decreased at lower temperatures [31,40]. Thus, to ensure the high control efficiency of pests, parasitoids may have to be released with greater frequency in warm regions because of the reduction in adult lifespan [31,35]. A low emergence rate may directly reduce the biological control performance, regardless of the parasitism capacity [37]. Our results show that this *T. remus* strain could be reared at temperatures ranging 20–32 °C, without negatively affecting the emergence rate, which may reflect the powerful adaptive capacity of *T. remus* over a wide temperature range. As female parasitoids could directly suppress populations of the target pest, a high female to male ratio may elevate the control efficiency of the parasitoid in biological control programs [21]. Our results reveal that the percentage of females was not clearly impacted by the temperature, and it was in agreement with previous reports [21,25].

Information regarding the thermal requirements of insect development will greatly influence mass-rearing programs [41]. The developmental threshold temperature and thermal constant for *T. remus* reared on eggs of *S. frugiperda* in our results (13.0 °C and 140.0 DD) differed from that of a study from Brazil, which reported that the corresponding values for *T. remus* reared on *S. frugiperda* were 14.9 °C and 125.6 DD, respectively [25]. These differences might correspond to the different geographic latitudes the parasitoid specimens were collected. Generally, insects originating from temperate regions have a lower development threshold, so they require more degree-days to complete development [42]. The developmental threshold temperature obtained from the current study was lower than that reported by Pomari et al. [25], which might be attributable to the higher geographic latitude of our population, which has a better tolerance to low temperatures. In this study, the slight differences in the thermal requirements of *T. remus* between the two host species tested might be due to the quality and amount of nutrients in the host egg, as well as the adaption of *T. remus* to specific eggs. Although the number of annual generations was predicted based on the thermal requirements [21], different field conditions need to be considered when developing a biological control program in certain regions. Moreover, information about the thermal requirements also provides a reference for the low-temperature storage of parasitoids.

Host preference is a key factor influencing the mass-rearing and application of parasitoids. Although parasitoid rearing on the factitious host is one of the essential steps in a biological control program, continuous rearing on the same host may affect its efficiency against the target pest after being released into the field [43]. These changes in host preference or parasitism are probably due to preimaginal conditioning occurring during parasitoid larval development [43]. Based on a previous study, the ability of parasitoids to distinguish hosts may be reduced or even lost after successive rearing on an alternative host [43]. However, according to Queiroz et al. [44], there was no obvious difference in the parasitism or host preference of *T. remus* on *S. frugiperda* eggs whether they were reared on *S. frugiperda* or *C. cephalonica* eggs. Although our results indicated that the *S. litura* eggs could be an alternative host of *T. remus*, in order to ensure the control efficiency against *S. frugiperda* in the field, it is necessary to further evaluate the host preference and parasitism when *T. remus* was successively reared on the *S. litura* eggs for several generations. Furthermore, if *T. remus* reared on *S. litura* eggs are released in a field where *S. litura* and *S. frugiperda* outbreaks concurrently, they may be controlled simultaneously. However, as *S. litura* is also an important agricultural pest, special attention should be paid so as to avoid bringing *S. litura* into the field when releasing parasitoids. In the application process, one of the effective strategies to improve the field practice of biological control programs is through the combined use of different biological control agents [6]. Investigations have revealed that predators are the most important species for controlling *S. frugiperda* in central Mexico, such as mite of the genus *Balaustium*, *Doru taeniatum* Dohrn and *Hippodamia convergens* Guérin-Meneville, and their control efficiency reached 63% [45]. Therefore, if different agents are used in combination, such as the combined use of parasitoids with predators, the control efficiency may be greatly improved. 

## 5. Conclusions

Our work proves that *T. remus* performed better on *S. litura* eggs in parasitism, as well as their suitability of progeny, compared with *S. frugiperda* eggs under all of the tested temperatures. Moreover, because there is little or no cannibalism, *S. litura* larvae can be reared in groups at a lower cost than *S. frugiperda*, suggesting that *S. litura* eggs might be more suitable as an alternative host for mass-rearing *T. remus* in China. This information on the biological characteristics and thermal requirements of *T. remus* provides important references for the large-scale reproduction and application of the parasitoid in biological control programs.

## Figures and Tables

**Table 1:** Results from a two-way ANOVA analysis on the effects of the temperature, host species, and their interactions on the development and biological characteristics of *T. remus*.

Parameters	T			H			T × H			Error
	F	df	*p*	F	df	*p*	F	df	*p*	
Egg-first instar larvae	3378.102	4	<0.0001	7.579	1	0.006	1.550	4	0.187	490
Second instar larvae	218.185	4	<0.0001	0.256	1	0.613	0.071	4	0.991	490
Prepupae	324.844	4	<0.0001	1.153	1	0.283	0.222	4	0.926	490
Pupae	13,226.875	4	<0.0001	27.431	1	<0.0001	39.061	4	<0.0001	490
Generation	43,620.894	4	<0.0001	8.006	1	0.005	42.906	4	<0.0001	490
Number of parasitized eggs	5.214	4	<0.0001	148.405	1	<0.0001	7.406	4	<0.0001	274
Lifespan	68.836	4	<0.0001	10.788	1	0.001	3.552	4	0.008	274
Percentage of female	1.935	4	0.105	0.080	1	0.777	1.611	4	0.172	274
Emergence rate	5.379	4	<0.0001	55.252	1	<0.0001	7.079	4	<0.0001	274

T—temperature; H—host species.

**Table 2:** Developmental duration of each of the stages of *T. remus* on the eggs of *S. frugiperda* or *S. litura* at constant temperatures.

Stage	Host	Development Duration (h) (Mean ± SE)					F	df	*p*
		20 °C	23 °C	26 °C	29 °C	32 °C			
Egg-first instar larvae	SF	148.3 ± 1.4 Aa	101.2 ± 0.9 Ab	71.0 ± 0.9 Ac	65.4 ± 0.7 Ad	54.3 ± 0.8 Ae	1530.136	4, 245	<0.0001
	SL	145.2 ± 1.2 Aa	101.0 ± 0.6 Ab	71.0 ± 0.8 Ac	64.1 ± 0.9 Ad	50.9 ± 0.7 Be	1889.782	4, 245	<0.0001
Second instar larvae	SF	40.1 ± 1.0 Aa	29.1 ± 0.8 Ab	25.2 ± 0.8 Ac	21.6 ± 0.7 Ad	18.4 ± 0.7 Ae	107.630	4, 245	<0.0001
	SL	39.8 ± 0.9 Aa	28.4 ± 0.8 Ab	25.2 ± 0.7 Ac	21.6 ± 0.7 Ad	18.2 ± 0.8 Ae	110.701	4, 245	<0.0001
Prepupae	SF	36.2 ± 1.0 Aa	26.6 ± 0.6 Ab	23.2 ± 0.8 Ac	12.7 ± 0.8 Ad	12.3 ± 0.6 Ad	166.942	4, 245	<0.0001
	SL	37.4 ± 1.1 Aa	27.5 ± 0.8 Ab	23.0 ± 0.8 Ac	13.1 ± 0.7 Ad	12.8 ± 0.6 Ad	158.575	4, 245	<0.0001
Pupae	SF	257.9 ± 0.7 Ba	189.8 ± 1.6 Ab	138.6 ± 0.6 Ac	101.7 ± 0.6 Bd	96.8 ± 0.6 Ae	5445.219	4, 245	<0.0001
	SL	263.8 ± 0.8 Aa	193.6 ± 1.1 Ab	134.1 ± 0.6 Bc	114.7 ± 0.6 Ad	92.4 ± 0.6 Be	8372.082	4, 245	<0.0001
Generation	SF	482.5 ± 0.7 Ba	346.7 ± 1.6 Ab	258.0 ± 0.6 Ac	201.4 ± 0.6 Bd	181.8 ± 0.6 Ae	18,101.270	4, 245	<0.0001
	SL	486.2 ± 0.8 Aa	350.5 ± 1.1 Ab	253.3 ± 0.6 Bc	213.5 ± 0.6 Ad	174.3 ± 0.6 Be	27,294.323	4, 245	<0.0001

SF—*S. frugiperda* eggs; SL—*S. litura* eggs. The same uppercase letter in the same column indicates that there was no significant difference in developmental duration between the two host species (*t*-test, *p* < 0.05); the same lowercase letters in the same row indicates that there was no significant difference in developmental duration among the five different temperatures (Tukey’s test, *p* < 0.05).

## Data Availability

The data presented in this study are available in article.
